# Asprosin and Neuregulin 4 in Obesity in Children

**DOI:** 10.3390/medicina61040723

**Published:** 2025-04-14

**Authors:** Ramazan Dulkadir, Gamze Turna Saltoglu, Ali Gunes

**Affiliations:** 1 Department of Pediatrics, Faculty of Medicine, Kirsehir Ahi Evran University, Kirsehir 40100, Turkey; 2 Department of Medical Biochemistry, Faculty of Medicine, Kirsehir Ahi Evran University, Kirsehir 40100, Turkey

**Keywords:** obesity, asprosin, neuregulin 4, child

## Abstract

*Background and Objectives*: Childhood obesity is a major problem in the nutritional aspect of childhood and is becoming increasingly important. The objective of this research was to examine the fluctuations in concentrations of asprosin and neuregulin 4, novel and significant adipokines, in obese pediatric individuals. *Materials and Methods*: In this study, comparisons were made between serum levels of asprosin and neuregulin 4, along with various anthropometric, biochemical, and hormonal parameters associated with obesity, among 40 children with obesity and 40 children with normal weight who sought medical attention at the Child Health and Diseases outpatient clinic of Kırşehir Training and Research Hospital between September 2021 and September 2022. *Results*: The study showed that of the 80 cases, 35 (43.8%) were male and 45 (56.2%) were female. The average BMI of the obese individuals was 27.27, with values ranging from 25.04 to 47.78. The serum levels of asprosin were similar between the two groups. Neuregulin 4 and HOMA-IR values exhibited statistically significant elevation in the obese group compared to the control group (*p* < 0.05). *Conclusions*: The research findings indicated that neuregulin 4 levels were greater in children with normal weight compared to those with children with obesity. Additional investigations are warranted to comprehensively grasp the impact of asprosin and neuregulin 4 on pediatric obesity.

## 1. Introduction

Obesity is a medical condition in which body fat accumulates due to an imbalance between calories consumed and calories expended, and it can lead to physical health problems [1]. Obesity rates among children and adolescents are increasing globally. As of 2016, there were 1.9 billion overweight and 650 million obese adults worldwide, with over 340 million overweight or obese children aged 5–19. Globally, the prevalence of overweight and obesity among children is approximately 18%, with variations observed based on race and ethnicity [2]. Children with a BMI percentile above 85 are classified as overweight, while those above 95 are classified as obese [3]. The primary risk factors contributing to obesity include poor dietary habits, physical inactivity, genetic predisposition, environmental factors, and socioeconomic status [4].

Asprosin is a recently discovered adipokine secreted by white adipose tissue, which exerts an orexigenic effect by stimulating hepatic glucose production during periods of energy demand [5,6]. Duerrschmid et al. have reported that asprosin functions as an orexigenic hormone, crossing the blood–brain barrier and directly activating orexigenic agouti-related peptide (AgRP) neurons through a cAMP-dependent mechanism. This activation triggers the sensation of hunger, leading to increased adiposity and weight gain [7]. Additionally, asprosin has been associated with insulin resistance and obesity [8,9]. Recent studies have demonstrated that asprosin is associated with glucose metabolism and the lipid profile, with elevated asprosin levels observed in individuals with impaired glucose control and correlation with insulin resistance in patients with polycystic ovary syndrome (PCOS) [6,9,10].

Neuregulin 4 (Nrg4), a recently discovered adipokine, is an epidermal growth-factor-like signaling molecule that plays a crucial role in maintaining the body’s energy balance [11,12]. Nrg4 has been associated with several metabolic disorders, including insulin resistance, obesity, nonalcoholic fatty liver disease (NAFLD), and diabetes [12,13].

Given that asprosin and Nrg4 have been shown to be closely associated with glucose and lipid metabolism, the investigation of their levels in individuals with childhood obesity is of significant importance. These adipokines could serve as key biomarkers for diagnosing obesity-related metabolic disorders and may represent potential targets in future therapeutic strategies.

Based on the above information, the aim of this study is to determine the levels of asprosin and Nrg4 in individuals with childhood obesity.

## 2. Method

Children with weight problems who applied to the Kırşehir Education and Research Hospital Child Health and Diseases polyclinic between September 2021 and September 2022 who did not have a chronic disease and met the study criteria were included in the patient group, and children who came for routine check-ups who did not have a weight problem or additional disease were included in the study as the control group. Our study is a preliminary study, and for this purpose, in this study, a total of 80 participants were included, with 40 (50.0%) children aged between 3 and 18 years in both the obese and control groups. Regarding gender distribution, 35 (43.8%) participants were male, and 45 (56.2%) were female.

The height, weight, and BMI percentiles of the patients, whose body weight and height were measured with a digital scale and stadiometer during the first examination, were re-evaluated according to charts appropriate for age and gender [14]. Those who were at the 95th percentile and above were considered obese [3]. The study included obese children under the age of 18 and healthy children without chronic diseases or obesity who attended routine check-ups. Patients with chronic diseases or additional complaints other than obesity were excluded from the study.

Approval for our study was obtained from the Ethics Committee of the Faculty of Medicine of Kirsehir Ahi Evran University (Approval no: 2021-13/148, Date: 3 August 2021). Informed consent was obtained from children and their parents who agreed to participate in the study.

### 2.1. Collection of Samples

Blood samples taken from patients diagnosed with children with obesity and healthy individuals were centrifuged at 1500× *g* for 10 min to obtain serum. The obtained serums were stored at −80 °C until the day of the study.

In these serum samples, in addition to asprosin and Nrg4 levels, parameters such as the lipid panel, fasting blood sugar, insulin level, ALT, AST and Homeostatic Model Assessment of Insulin Resistance (HOMA-IR) index were also analyzed. The healthy control group consisted of children who voluntarily visited the hospital for general check-ups and had no comorbidities. Additionally, the body mass index (BMI) was determined by an expert physician during the outpatient clinic examination.

### 2.2. Determination of Asprosin and Neuregulin 4 Levels by Elisa Method

Serum asprosin levels were measured using the Human Asprosin ELISA kit (Uscn Life Science Inc., Wuhan, China), while serum neuregulin 4 levels were assessed using the Human Neuregulin 4 ELISA kit (Elabscience Biotechnology Inc., Wuhan, China). Both assays were conducted in accordance with the manufacturers’ protocols, ensuring adherence to the specified instructions to maintain accuracy and reliability. The concentrations of asprosin and neuregulin 4 were determined using the SPECTROstar Nano microplate reader (BMG LABTECH, Ortenberg, Germany). Samples were analyzed in duplicate to ensure the consistency and reliability of the results.

### 2.3. Statistical Analysis

The data were analyzed using the IBM SPSS Statistics Standard Concurrent User V 26 software package (IBM Corp., Armonk, New York, NY, USA). Descriptive statistics were presented as the number of units (n), percentage (%), mean ± standard deviation ($\bar{x}$ ± ss), median (M), minimum (min), and maximum (max) values. The normal distribution of numerical variables was assessed using the Shapiro-Wilk normality test. For comparing differences between two groups, the “Independent Two Sample *t*-test” was utilized when the data were normally distributed. In cases where the data did not follow a normal distribution, the “Mann–Whitney U test” was employed. Additionally, the “Chi-square test” was applied for analyzing categorical data. Sensitivity and selectivity rates were calculated by drawing the ROC curve from diagnostic test applications for the Nrg4 value. *A p* value of <0.05 was considered statistically significant.

## 3. Results

The gender distribution between the obese and control groups was similar (*p* > 0.05). No significant relationship was found between gender and asprosin and Nrg4 (Table 1). The obese group has statistically higher values for age, height, and weight compared to the control group (*p* = 0.006, *p* = 0.022, *p* < 0.001, respectively). The body mass index (BMI) was significantly higher in the obese group compared to the control group (*p* < 0.001). HDL levels were statistically higher in the control group than in the obese group (*p* = 0.015). No statistically significant differences were found between the groups for LDL and cholesterol levels. Triglyceride levels were statistically significantly higher in the obese group compared to the control group (*p* = 0.008). ALT levels in the obese group were statistically higher than those in the control group (*p* = 0.005). AST levels in the obese group were statistically lower than those in the control group (*p* = 0.017). Insulin levels were higher in the obese group compared to the control group, and this difference was statistically significant (*p* < 0.001). No statistically significant difference was found between the groups for asprosin levels. Nrg4 and HOMA-IR values were higher in the obese group compared to the control group, and this difference was statistically significant (*p* = 0.028, *p* < 0.001, respectively) (Table 2).

**Figure 1:**
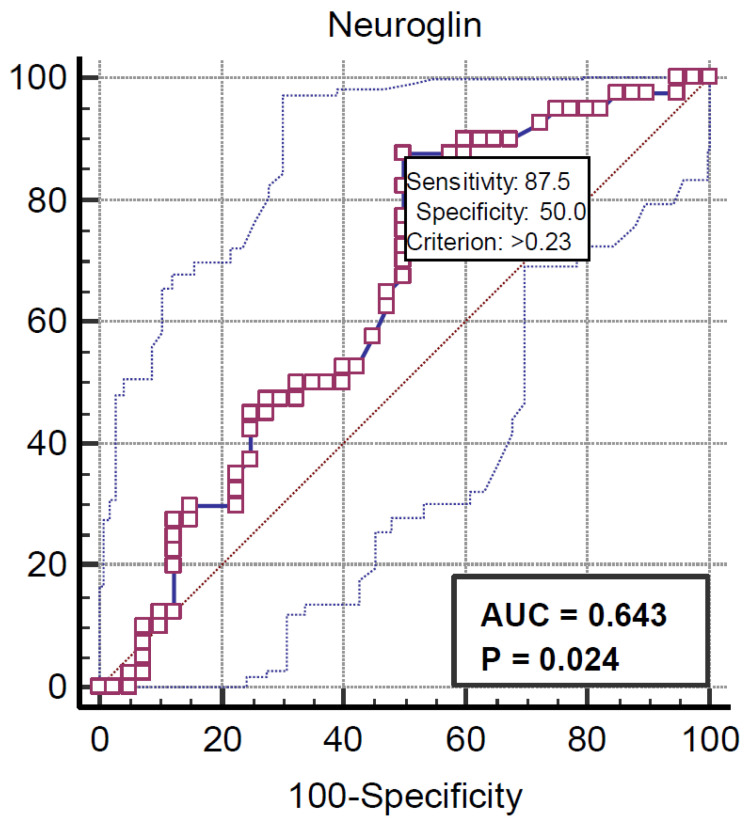
ROC curve for neuregulin 4 in children with obesity. ROC: receiver operating characteristic, AUC: area under the ROC curve, P: significance level (area = 0.5).

Neuregulin’s relationship with children with obesity was directly proportional on the ROC curve and was determined with 87.5% sensitivity and 50% specificity (Figure 1).

## 4. Discussion

The findings of this study demonstrate significant differences in biochemical parameters between children with obesity and those with normal weight. The obese group showed significantly higher values in anthropometric measures such as age, height, weight, and body mass index (BMI) compared to the control group. While HDL levels were higher in the control group, triglyceride levels were significantly higher in the obese group. Furthermore, ALT levels were higher in the obese children compared to the control group, while AST levels were lower in the obese group. Insulin and HOMA-IR levels were significantly higher in the obese group compared to the control group, indicating insulin resistance associated with obesity. Although no significant difference was found in asprosin levels between the groups, Nrg4 levels were significantly higher in the obese group. Additionally, ROC analysis revealed a direct relationship between Nrg4 and obesity, suggesting a potential role of this marker in the diagnosis of obesity. These results may contribute to a better understanding of the biochemical changes associated with obesity and the development of biomarkers for obesity management.

Children with obesity are at increased and later risk of long-term chronic diseases such as type 2 diabetes, dyslipidemia, and carotid atherosclerosis, as well as fatal and non-fatal cardiovascular events in adulthood, compared with their non-obese peers [15]. It is also known that the majority of individuals who were obese in childhood continue their lives as obese in adulthood [16]. Therefore, it can be said that childhood obesity is not limited to itself but also paves the way for chronic diseases. Therefore, it is extremely important to determine all factors that cause childhood obesity.

In their study, Long et al. observed that asprosin levels were lower in children with obesity compared to their normal-weight counterparts [8]. In our study, although there was no statistically significant difference between the groups, the asprosin levels in the obese group were found to be relatively low compared to the control group. This finding may be attributed to the cases within the obese group being in the early stages of obesity. Future studies may be warranted to repeat this investigation.

Contrary to these findings, Wang et al. reported that serum asprosin levels significantly decreased in patients who underwent bariatric surgery and lost weight, compared to pre-surgical levels [17]. Furthermore, they stated that serum asprosin levels were the only independent predictor of the percentage of weight loss following these surgeries [17]. In a study by Ugur et al. comparing serum asprosin levels between obese patients and healthy controls, it was found that the amount of asprosin increased from the lean group to the obese group [18]. Studies have not yet clarified whether there is a difference between genders in asprosin levels. However, a study conducted in children with obesity found that asprosin levels were significantly lower in obese boys compared to obese girls. This difference was not observed between non-obese boys and girls [4]. In our study, no significant difference was found between gender and asprosin and Nrg4 in the obese and control groups. As can be seen from these results, although contradictory findings have been reported, serum asprosin levels appear to be closely associated with metabolism [19].

It has been suggested that Nrg4 may support energy metabolism by increasing energy expenditure and fuel oxidation as well as promote the production of metabolically beneficial adipokines [20]. Furthermore, it has been stated that Nrg4 may improve obesity by modulating the process of angiogenesis in adipose tissue [20]. Studies investigating serum Nrg4 levels in children are quite limited. In this regard, our study holds significant value. In our study, serum Nrg4 levels were found to be significantly higher in children with obesity compared to normal-weight controls. It has been reported that the excessive secretion of Nrg4 prevents weight gain and fatty liver induced by a high-fat diet [21]. Wang et al. found significantly lower Nrg4 levels in obese children with non-alcoholic fatty liver disease compared to the control group [22]. Cao et al. found higher Nrg4 levels in obese adolescents with PCOS compared to the non-PCOS group [23]. Martinez et al. also identified that Nrg4 may be associated with insulin resistance [24].

Nevertheless, there are some limitations in our study. First, the literature on asprosin and Nrg4 levels in obese children is relatively limited, which makes it challenging to comprehensively compare our findings with those of other studies. Additionally, our sample size is relatively small, which may limit the generalizability of the results. Larger sample sizes would enhance the reliability of the data and provide a more in-depth investigation into the role of asprosin and Nrg4 in the pathogenesis of obesity.

## 5. Conclusions

Consequently, our study stands out as one of the rare studies demonstrating that Nrg4 levels are higher in children with obesity compared to their normal-weight counterparts. Although the effects of asprosin and Nrg4 are not fully understood, both have been shown to be associated with various chronic diseases; however, further research is needed to confirm their roles in causing obesity in children.

## Figures and Tables

**Table 1:** Relationship between asprosin and neuregulin and gender.

Variables	Group	N	Mean	Sd	t	*p* Value
Asprosin	Female Male	45 35	18,669 0.8366	120,775 0.196	0.872	0.08
Nrg4	Female Male	45 35	1033 1694	1876 6139	−0.689	0.246

**Table 2:** Comparison of clinical features by group.

	Groups		Test Statistics	
	Obese	Control	Test Value	*p* Value
Gender, n (%) Male Female	21 (60.0) 19 (42.2)	14 (40.0) 26(57.8)	χ^2^ = 2.489	0.115
Age (years) M (min–max)	11 (6–18)	9 (3–16)	z = 2.745	0.006
Height (cm) M (min–max)	149.5 (124.0–170.0)	138.5 (106.0–182.0)	z = 2.291	0.022
Weight (kg) M (min–max)	64.65 (13.02–110.0)	32.75 (15–65)	z = 5.692	<0.001
Body Mass Index (kg/m^2^) M (min–max)	27.27 (25.04–47.78)	17.96 (12.63–27.69)	z = 7.477	<0.001
HDL (mg/dL) M (min–max)	44 (33–78)	50 (30–81)	z = 2.426	0.015
LDL (mg/dL) M (min–max)	91 (58–302)	79 (38–151)	z = 1.734	0.083
Cholesterol (mg/dL) M (min–max)	158.5 (113–369)	154 (97–219)	z = 0.742	0.458
Triglyceride (mg/dL) M (min–max)	96.50 (48–236)	70 (37–277)	z = 2.649	0.008
VLDL (mg/dL)	21.5 (9–47)	15.3 (7–55)	z = 1.231	0.162
Glucose (mg/dL) $\bar{x}$ ± sd	96.75 ± 8.79	93.55 ± 9.64	t = 1.531	0.130
ALT (U/L) M (min–max)	19.5 (5–65)	15 (7–161.0)	z = 2.814	0.005
AST (U/L) M (min–max)	26 (14–52)	30 (13–209)	z = 2.382	0.017
Insulin M (min–max)	12 (3–24)	6 (2–32)	z = 4.050	<0.001
Asprosin (ng/mL) M (min–max)	0.78 (0.46–1.23)	0.82 (0.55–2.65)	z = 0.760	0.447
Neuregulin 4 (ng/mL) M (min–max)	0.45 (0.04–5.24)	0.28(0.02–36.74)	z = 2.194	0.028
HOMA-IR M (min–max)	3.10 (0.70–6.10)	1.30 (0.40–6.90)	z = 3.801	<0.001

$\bar{x}$: Mean, sd: standard deviation, M: median, t: Independent Sample *t*-test, z: Mann–Whitney *U* test.

## Data Availability

The original contributions presented in this study are included in the article. Further inquiries can be directed to the corresponding author.
